# Selectivity Profiling and Biological Activity of Novel β-Carbolines as Potent and Selective DYRK1 Kinase Inhibitors

**DOI:** 10.1371/journal.pone.0132453

**Published:** 2015-07-20

**Authors:** Katharina Rüben, Anne Wurzlbauer, Agnes Walte, Wolfgang Sippl, Franz Bracher, Walter Becker

**Affiliations:** 1 Institute of Pharmacology and Toxicology, RWTH Aachen University, Aachen, Germany; 2 Department of Pharmacy—Center for Drug Research, Ludwig Maximilian University, Munich, Germany; 3 Institute of Pharmacy, Martin Luther University Halle-Wittenberg, Halle, Germany; IGBMC/ICS, FRANCE

## Abstract

DYRK1A is a pleiotropic protein kinase with diverse functions in cellular regulation, including cell cycle control, neuronal differentiation, and synaptic transmission. Enhanced activity and overexpression of DYRK1A have been linked to altered brain development and function in Down syndrome and neurodegenerative diseases such as Alzheimer’s disease. The β-carboline alkaloid harmine is a high affinity inhibitor of DYRK1A but suffers from the drawback of inhibiting monoamine oxidase A (MAO-A) with even higher potency. Here we characterized a series of novel harmine analogs with minimal or absent MAO-A inhibitory activity. We identified several inhibitors with submicromolar potencies for DYRK1A and selectivity for DYRK1A and DYRK1B over the related kinases DYRK2 and HIPK2. An optimized inhibitor, AnnH75, inhibited CLK1, CLK4, and haspin/GSG2 as the only off-targets in a panel of 300 protein kinases. In cellular assays, AnnH75 dose-dependently reduced the phosphorylation of three known DYRK1A substrates (SF3B1, SEPT4, and tau) without negative effects on cell viability. AnnH75 inhibited the cotranslational tyrosine autophosphorylation of DYRK1A and threonine phosphorylation of an exogenous substrate protein with similar potency. In conclusion, we have characterized an optimized β-carboline inhibitor as a highly selective chemical probe that complies with desirable properties of drug-like molecules and is suitable to interrogate the function of DYRK1A in biological studies.

## Introduction

Protein kinases are pharmacologically tractable proteins that have key roles in most, if not all, cellular signalling pathways. Although protein kinases have become one of the most intensively pursued classes of drug targets, selective inhibitors suitable for functional experiments exist only for a fraction of the human kinome [1,2]. Although RNA interference and genetic knockout techniques provide valuable functional information, small molecules can inhibit kinase catalytic activity without perturbing the function of other domains that have independent functions in many protein kinases [3]. Thus, high quality chemical probes are needed for the functional characterisation of the >500 human protein kinases and their evaluation as potential drug targets [4–6].

Protein kinases of the DYRK family are dual specificity kinases that phosphorylate substrates only on serine or threonine residues but autophosphorylate on tyrosine, which is an essential step for full activation of the enzymes [7–9]. DYRK1A has attracted increasing interest as a potential drug target due to its role in the pathology of Down syndrome and the proposed involvement in neurodegenerative diseases and cancer (for reviews, see [10–14]). Owing to the localisation of the human *DYRK1A* gene on chromosome 21, the over-activity of DYRK1A that results from the increased dosage of the *DYRK1A* gene is thought to contribute to the neurological abnormalities associated with Down syndrome [15]. The function of DYRK1A in neurogenesis and neuronal differentiation is well supported by evidence from cell culture, transgenic mouse models and human disease [11,16–17]. At least in mice, DYRK1A overexpression results also in postnatal electrophysiological and cognitive alterations, suggesting that this phenotype might be amenable to pharmacological intervention [18]. Indeed, the effects of DYRK1A overexpression on brain function in transgenic mice can be partially rescued in adult animals by downregulation or inhibition of DYRK1A [19–21].

DYRK1A is a pleiotropic kinase that is ubiquitously expressed and phosphorylates many proteins unrelated to neuronal differentiation and function [11,22–24]. The participation of DYRK1A in the regulation of many cellular processes, such cell survival, quiescence, mRNA splicing, endocytosis and transcriptional regulation is often supported by the effects of kinase inhibitors. DYRK1B is a paralogous kinase closely related with DYRK1A (85% identical amino acids in the catalytic domain) and is overexpressed in certain cancer types, where it favours the arrest of cells in a quiescent state to allow cellular repair [25–26]. Interestingly, a gain-of-function point mutation in *DYRK1B* has been identified as causative for a familial form of the metabolic syndrome [27]. A highly selective small-molecule inhibitor of DYRK1A and DYRK1B will be instrumental in defining the physiological substrates and downstream effects that are regulated by these kinases.

The plant alkaloid harmine is one of the most potent and selective DYRK1A inhibitors presently available [13–14,28]. Enzymatic studies and the analysis of the DYRK1A/harmine cocrystal have characterized harmine as an ATP competitive inhibitor that binds to the active conformation of the kinase domain (type I inhibitor) [29–30]. Nevertheless, harmine proved to be highly selective for DYRK1A and DYRK1B in a kinome screen [31]. Importantly, harmine inhibits DYRK1A-dependent phosphorylation events in cultivated cells with similar potency as the recombinant kinase, which indicates that harmine is cell-permeable and can inhibit DYRK1A at normal cellular ATP concentrations [28]. Therefore, harmine is frequently used to scrutinize the presumed role of DYRK1A in cellular processes [14]. However, the use of harmine as a chemical probe for DYRK1A and DYRK1B is compromised by the fact that it is also a high affinity inhibitor of monoamine oxidase A (MAO-A). Actually, harmine is commonly used as a tracer in positron emission tomography (PET) studies for the specific visualization and quantification of MAO-A in human brain [32].

Based on the already favorable properties of harmine as a kinase inhibitor, we have synthesized a series of substituted harmine analogs with reduced MAO-A inhibitory action. The present study aims to select from this panel the best chemical probe for DYRK1A. We have extensively studied the most promising new β-carbolines for their kinase selectivity and their efficacy in cell based assays. These studies identified compounds with minimal (AnnH31) or absent (AnnH75) effect on MAO-A activity and very high kinase selectivity that are potently suppress the phosphorylation of DYRK1A substrates in cultured cells.

## Results and Discussion

### Kinase selectivity of the novel β-carboline DYRK1A inhibitors

An extensive panel of new harmine analogs was designed and synthesized in order to develop a new DYRK1A inhibitor without MAO-A inhibitory activity (unpublished work). From this series, we selected those compounds for the present analysis that showed comparable or better inhibition of DYRK1A than harmine (> 60% inhibition at a concentration of 1 μM) and did not inhibit MAO-A (<5% at 1 μM). As an exception, AnnH31 was included although it weakly inhibited MAO-A in the primary screen (25% inhibition at 1 μM).

**Fig 1:**
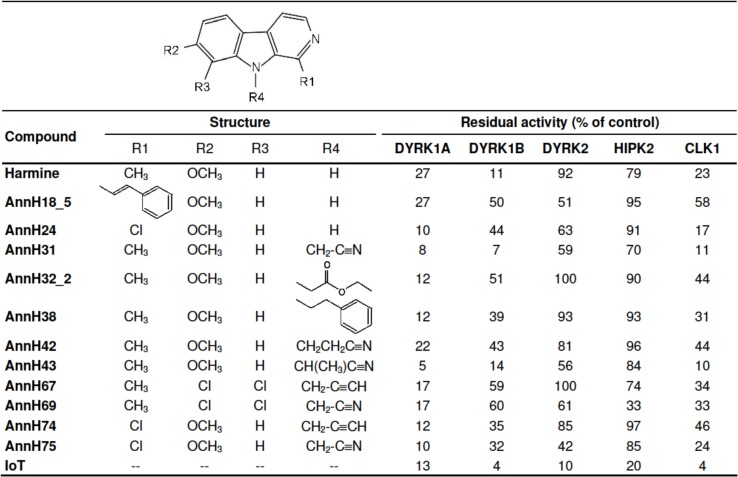
Inhibition of DYRK1A and related kinases by selected β-carbolines. Kinase activities are given as the means of at least 3 measurements in the presence of 1 μM of the compounds (10 μM in HIPK2 assays) and are expressed as the percentage of the uninhibited control (Kinase-GLO assay). 5-iodotubercidin (IoT) served as a structurally unrelated control compound that inhibits all tested kinases.

First, the new β-carbolines were examined for selectivity against kinases that are typical targets of other DYRK1A inhibitors, *i*.*e*. DYRK1B, DYRK2, HIPK2 and CLK1. Overall, most compounds showed a similar selectivity profile towards these kinases (Fig 1). The more potent DYRK1A inhibitors also inhibited DYRK1B, although slightly less efficiently than DYRK1A. In contrast to 5-iodotubercidin, which we used as a reference compound, the β-carbolines inhibited DYRK2 and HIPK2 only weakly. Similar to other DYRK1A inhibitors, most compounds were also active against CLK1 [14,31].

To further characterize the selectivity of three promising compounds, we determined IC_50_ values for the inhibition of MAO-A and DYRK1A. In this experiment we measured DYRK1A activity by a radioactive assay, which is considered the gold standard for measuring kinase activities [33]. This assay identified AnnH31 as the most potent DYRK1A inhibitor with an IC_50_ of 81 nM, followed by AnnH75 and AnnH43 (Table 1). N9-substituted harmine derivatives (AnnH31 and AnnH43) were less active towards MAO-A. The N9-cyanomethyl derivative of harmine, AnnH31, inhibited MAO-A with an IC_50_ of 3.2 μM, which translates to 40 fold selectivity for DYRK1A over this off-target. The concept of using N-alkylation of harmine to reduce MAO-A inhibition has previously been exploited by Drung *et al*. [34], who showed that N9-heptyl harmine inhibits MAO-A much less potently than DYRK1A (13% vs. 85% inhibition at a concentration of 1 μM). Eventually, we achieved the complete elimination of MAO-A inhibition by generating the 1-chloro analog of AnnH31, AnnH75. Considering our aim to achieve maximal selectivity for DYRK1A over MAO-A, we opted for AnnH75 as the most useful compound. Regarding the intended use in animal experiments, we also speculated that the chlorination of ring A might increase metabolic stability of AnnH75.

**Table 1:** Inhibition of DYRK1A and MAO-A by selected β-carbolines.

Compound	Inhibition of DYRK1A^a^	Inhibition of MAO-A^a^
	IC_50_, nM	IC_50_, nM
Harmine	(33–80)^b^	107
AnnH31	81	3,240
AnnH43	202	5,390
AnnH75	181	>10,000

^a^ Calculated by curve fitting from n = 3–5 independent concentration response curves shown in Fig. A in S1 File.

^b^ Range of published values [28,29,56].

### Docking analysis

**Fig 2:**
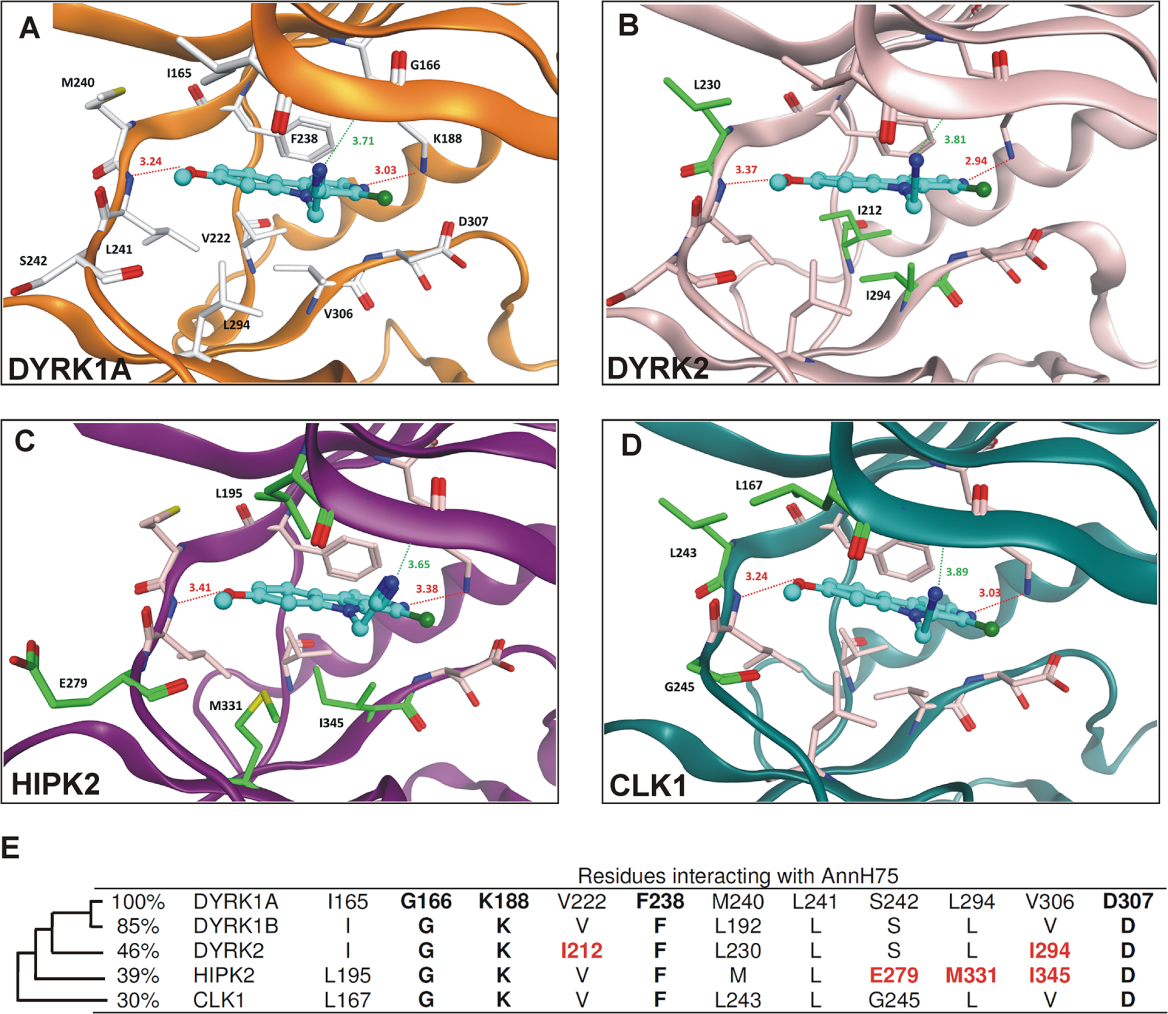
Analysis of kinase inhibitor interactions. **A-D**, Predicted binding modes of AnnH75 at DYRK1A and related kinases. The inhibitor is colored cyan and kinases are depicted as ribbon structures. Only relevant amino acid residues in the ATP binding pocket are shown for clarity. In B-D, residues different from DYRK1A are colored green and labeled. The distances of the two hydrogen bonds (red line) and the distance between the cyano group and the glycine of the P-loop (G166 in DYRK1A) are given in Angstrom. (DYRK1A, PDB ID 3ANR; DYRK2 PDB ID 4AZF; HIPK2, homology model, CLK1, PDB ID 2VAG). **E**, Comparison of amino acid residues relevant for AnnH75 binding. In the left, the relationship of the kinases is illustrated by the sequence identity of their catalytic domains. Residues generally conserved in protein kinases (G166, K188, D307) and the gatekeeper residue (F238) are highlighted by bold print. Residues different from DYRK1A are shown with their position in the sequence. Differences correlating with kinase resistance to AnnH75 are highlighted in red.

To rationalize the observed *in vitro* inhibition data and to analyse the interaction with the selected kinases we performed a molecular docking study. As observed in the X-ray structure of DYRK1A complexed with harmine, the analog AnnH75 could be docked in a similar way to DYRK2, HIPK2 and CLK1 (Fig 2). AnnH75 interact with the residues of the ATP binding pocket and is involved in two hydrogen bonds–one to the hinge region (backbone NH of L241) and one to the conserved K188 (Fig 2A). The cyanomethyl substituent R4 is buried under the P-loop of DYRK1A and makes favourable van-der-Waals interaction with G166 (distance shown in Fig 2). The same binding mode was observed for DYRK1B where only one residue of the binding pocket differs from DYRK1A (L192 instead of M240 in DYRK1A) (Fig. B in S1 File). The inhibitor binding site of DYRK2 is also similar to that of DYRK1A with just three residues that are different (Fig 2B). V222 in DYRK1A is changed to an isoleucine in DYRK2 (I212), while the DYRK1A hinge residue M240 is changed to a leucine in DYRK2 (L230), which slightly decreases the hydrophobicity of the pocket. The residue that precedes the DFG motif (V306 in DYRK1A and I294 in DYRK2) appears to be more important. I212 and I294 in DYRK2 are more bulky than the corresponding residues in DYRK1A and change the size of the entrance of the ATP pocket, which seems to be less favourable for the N9-substituted harmine analogs studied in the current work. However, this point cannot be demonstrated with the simple docking models presented here.

The HIPK2 ATP binding pocket shows the largest structural deviation compared to DYRK1A (Fig 2C and 2E). As a consequence, the van-der-Waals interaction between M331 in HIPK2 and the carboline ring of AnnH75 is less favourable compared to L294 in DYRK1A. Interestingly, the binding pocket of CLK1 is more similar to DYRK1A, although CLK1 is a more distant member of the family. Two residues in contact with the inhibitor are different: M240 in DYRK1A is changed to L243 in CLK1 (L241 in CLK4) and I165 in DYRK1A is changed to L167 (L165 in CLK4), respectively (Fig 2D and 2E). The third difference (S242 in DYRK1A changed to G245 in CLK1) is not in direct contact with the inhibitor. Due to the high similarity of the ATP binding pocket (shape and hydrophobicity), the docking pose of AnnH75 for CLK1 (and CLK4) is nearly identical to the one observed for DYRK1A/B and explains the observed inhibitory activity of AnnH75 for CLK1.

### Kinome profiling

**Fig 3:**
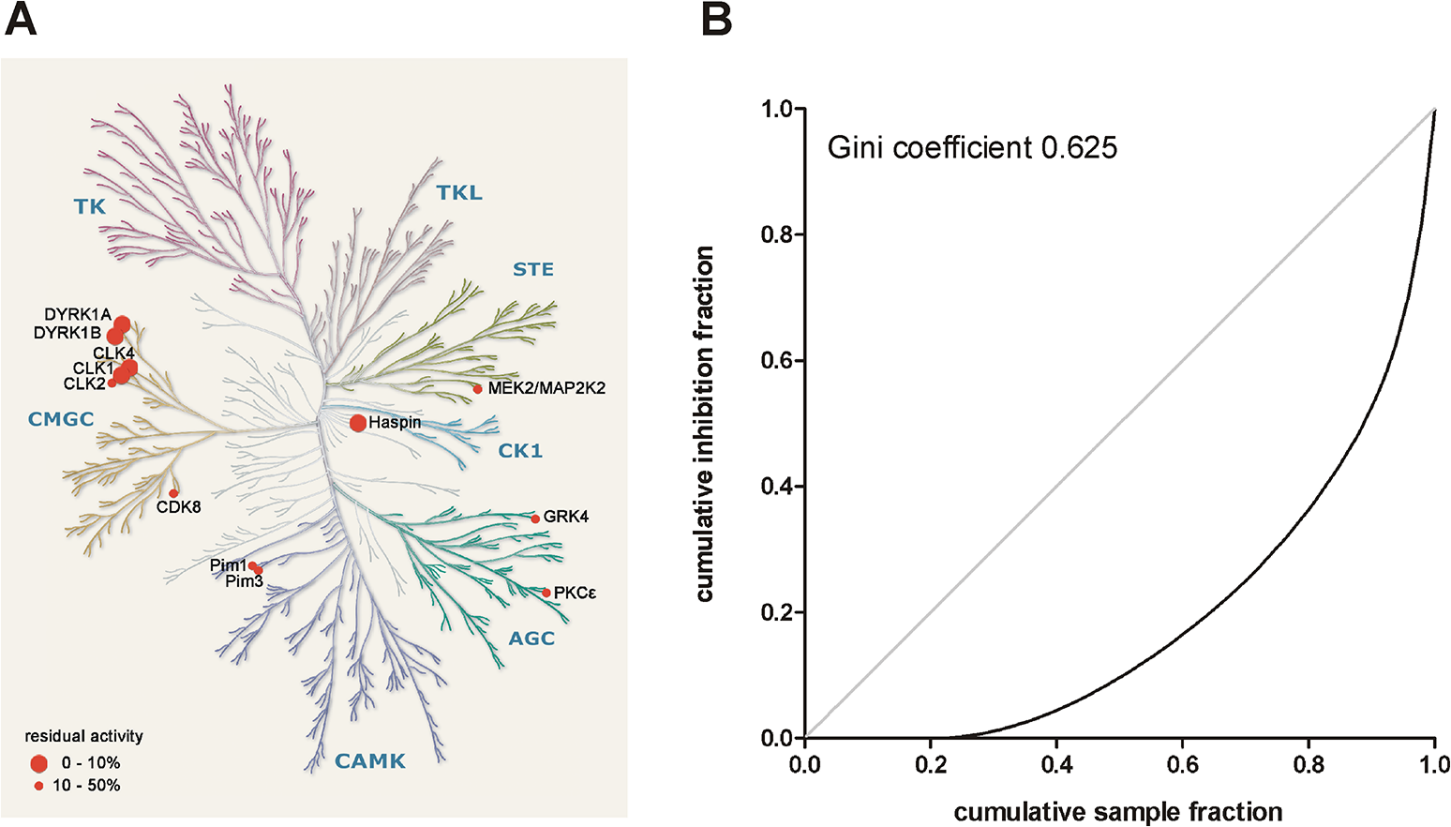
Selectivity profile of AnnH75. AnnH75 was profiled at a concentration of 1 μM against a panel of 300 protein kinases (see Table A in S1 File for the complete results). **A**, Target kinases inhibited by more than 50% are indicated. The kinome dendrogram was adapted and is reproduced courtesy of Cell Signaling Technology. **B**, Calculation of the Gini coefficient as a measure of kinase selectivity. The Lorenz curve illustrates the degree to which the total inhibitory activity of a compound (i.e. the sum of inhibition of all tested kinases) is equally distributed among all tested kinases (bisector line, Gini coefficient of 0) or directed towards a single kinase (maximal selectivity, a Gini coefficient of 1).

The selectivity of AnnH75 was profiled against a panel of 300 kinases at a concentration of 1 μM (Table A in S1 File). These activity assays identified CLK1, CLK4, haspin (gene symbol GSG2), DYRK1A and DYRK1B as the kinases most strongly inhibited by AnnH75 (Fig 3A). The cross-kinase activity towards CLK1/4 is typical for DYRK1A/B inhibitors with published selectivity data, suggesting that these kinases share a similar geometry of their ATP binding pockets (Table 2). Nevertheless, two new compounds derived from different scaffolds were recently reported to exhibit 100-fold selectivity for DYRK1A over CLK1 [35–36]. These compounds could not be included in Table 2 because kinome profiling data are not available. Maximal specificity is a desirable goal for a chemical probe, whereas polypharmacology may be acceptable for a drug [6]. In fact, the combined DYRK1A/CLK1 inhibitory activity has been proposed to be advantageous to correct the dysregulated alternative splicing of tau mRNA, because both kinases are thought to favour the generation of pathogenic tau species [37]. On the other hand, the issue of potentially redundant functions of the CLKs is not yet sufficiently clear, so that the relative resistance of CLK2 and CLK3 to the inhibitors may allow these kinases to compensate for the effects of CLK1/4 inhibition.

**Table 2:** Kinome selectivity of AnnH75 and published DYRK1A inhibitors.^a^

	Residual activity (% of control)							
	AnnH75 (1 μM)	Harmine (1μM)	Harmine (10 μM)	L41 (10 μM)	Leucett-amine B (10 μM)	INDY (10 μM)	LDN-211898 (10 μM)	Thiophene 29 (5 μM)
**Kinase**								
CLK1	0	-	0	0	2	1	0	4
CLK2	43	10	3	6	10	12	1	28
CLK3	74	-	42	2	73	30	63	88
CLK4	0	-	2	0	2	0	-	0
DYRK1A	8	2	0	0	2	0.23 μM^b^	0	10
DYRK1B	8	-	2	4	11	0.24 μM^b^	2	0
DYRK2	68	35	61	1	57	3	14	7
DYRK3	57	31	-	-	-	0	3	34
DYRK4	94	-	-	-	-	13	-	87
HIPK1	91	119	14	1	34	64	59	96
HIPK2	87	68	34	11	60	-	47	-
HIPK3	88	118	44	4	45	-	51	-
HIPK4	88	-	90	44	91	-	72	93
IRAK1	95	-	66	3	100	-	47	-
PIM1	41	74	85	15	73	2	9	78
PIM2	107	101	43	41	82	-	36	93
PIM3	44	74	78	27	71	-	11	-
Haspin	5	0.59 μM^c^	-	-	-	-	0	-
**Number of kinases**	300	117	402	402	402	66	292	102
**Reference**		[45]	[31]	[31]	[31]	[30]	[38]	[57]
**Gini score**	0.625	0.628	0.843	0.673	0.862	0.342	0.652	0.741
**Selectivity score**	0.04	0.04	0.06	0.09	0.02	0.26	0.16	0.1

^a^ Structures are shown in Fig. D in S1 File.

^b^ IC_50_ values are given because %-activity values are not given in the reference.

^c^ IC_50_ value taken from Cuny et al. [38]

AnnH75 and harmine lack cross-reactivity for the related kinases DYRK2, DYRK3 and HIPK1-3, which presents a clear advantage over L41, INDY and LDN-211898. Thiophene 29 does not inhibit the HIPKs, but targets DYRK2 (Table 2).

The DYRK, HIPK and CLK families are neighbours in the CMGC branch of the phylogenetic kinome tree (Fig 3A). The only other off-target of AnnH75 was haspin (gene name GSG2), an atypical protein kinase with limited amino acid sequence similarity to the DYRK/CLK families. Dual inhibition of DYRKs and haspin has previously been observed for other β-carbolines [38] but also for structurally divergent inhibitors such as acridine compounds [39], 5-iodotubercidin [40] and the anthrapyrazolone SP600125 [41]. The best characterized function of haspin is the phosphorylation of Thr3 in histone H3 during mitosis [42]. Knockdown of haspin in human cell lines has been reported to cause mitotic arrest [43].

To compare the overall kinase inhibitor selectivity of AnnH75 and other inhibitors, we calculated a selectivity score (S) for each compound by dividing the number of target kinases (< 50% residual activity) by the total number of tested kinases. As a second measure of selectivity, we determined the Gini coefficient, which does not depend on defining an arbitrary hit threshold (Fig 3B) [44]. According to these metrics, AnnH75 is as selective as harmine and superior to INDY [30,45]. The higher Gini values of L41 and thiophene 29 are likely due to the higher inhibitor concentrations used in these screens, as the Gini coefficient is highly dependent on the inhibitor concentration [44]. This is exemplified by the data sets for 1 μM and 10 μM harmine in Table 2.

### Cytotoxicity

To evaluate the usability of the new DYRK1A inhibitors in cell culture experiments, we analyzed the effect of AnnH75 and other harmine analogues on the viability of three different clonal cell lines, HeLa cells and the neuronal cell lines PC12 and SH-SY5Y. Viability assays showed that treatment with up to 10 μM AnnH75 for 3 days caused minimal cytotoxicity in HeLa and PC12 cells (Table 3), whereas AnnH24, AnnH31 and AnnH43 reduced cell viability when used at high concentrations. We have previously found cytotoxic effects of harmine at concentrations of >3 μM in HeLa and HEK293 cells [28]. The SH-SY5Y neuroblastoma cell line was more sensitive towards all tested compounds than the other two cell lines. Of note, AnnH75 was used at concentrations sufficient for maximal inhibition of haspin without markedly affecting the viability of HeLa and PC12 cells. In contrast, the haspin inhibitor CHR-6494 caused dose-dependent reduction of viability of three different cell lines by blocking cell cycle progression and inducing mitotic spindle abnormalities with large defects in chromosomal alignment [46]. The lack of toxicity of AnnH75 in the viability assay suggests haspin inhibition does not inevitably result in mitotic arrest, since the XTT assay is sensitive the cell number and thus to changes in the proliferation rate.

**Table 3:** Cytotoxicity of selected compounds.^a^

	HeLa			PC12			SY5Y		
	1 μM	3 μM	10 μM	1 μM	3 μM	10 μM	1 μM	3 μM	10 μM
AnnH24	97	96	72	95	90	81	75	66	45
AnnH31	93	80	55	89	88	70	81	88	38
AnnH43	91	80	66	88	88	90	61	68	45
AnnH75	100	93	92	94	100	95	89	73	43
IoT^b^	67	16	18	62	17	5	13	10	15
Staurosporine^c^	8			18			6		

^**a**^ Viability of treated cells (HeLa, PC12 and SH-SY5Y) is given in percent relative to untreated control cells (means of two experiments with triplicate measurement).

^b^ 5-Iodotubercidine (IoT) is a DYRK/CLK inhibitor that inhibits viability by acting on an off-target (adenylate kinase).

^c^ Staurosporine is known to induce apoptosis and served as a positive control.

### Inhibition of DYRK1A activity in cell culture

**Fig 4:**
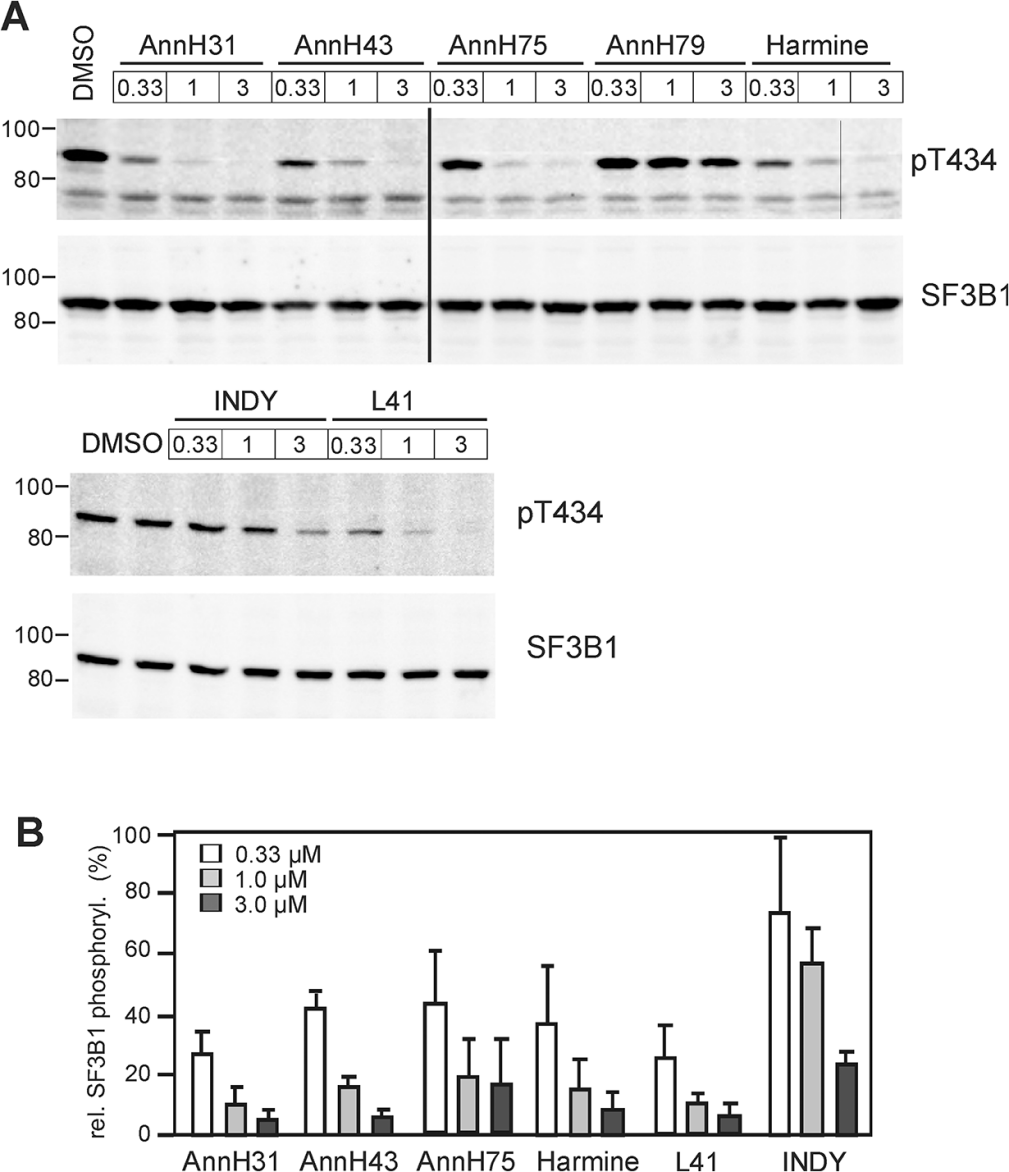
Inhibition of SF3B1 phosphorylation by DYRK1A in HeLa cells. HeLa cells expressing GFP-SF3B1-NT were treated with the indicated compounds for 18 h. The phosphorylation state of SF3B1 was determined by immunoblotting with pT434 antibody, and the results were normalized to the total amount of SF3B1 immunoreactivity. **A**, Representative western blots. AnnH79 is a harmine analogue that does not inhibit DYRK1A and was used as negative control. The vertical line indicates where irrelevant lanes were deleted from the final image. **B,** The column diagram summarizes the quantitative evaluation of 3–6 experiments for each compound (means + SD).

To evaluate the new harmine analogues for their capacity of inhibiting DYRK1A in cell culture experiments, we compared the effect of AnnH75 and other select compounds on the DYRK1A-catalysed phosphorylation of splicing factor 3b1 (SF3B1). Phosphorylation of SF3B1 on Thr434 closely reflects the cellular activity of endogenous DYRK1A and provides a useful measure for the cellular efficacy of DYRK1A inhibitors [28]. HeLa cells were transiently transfected with a GFP-SF3B1 expression vector and treated with three of the new β-carbolines (AnnH31, AnnH43, AnnH75) in parallel with three established DYRK1A inhibitors (harmine, L41, INDY). Western blot analysis showed that the new inhibitors reduced the phosphorylation of SF3B1 with potencies similar to harmine and L41 and were more active than the benzothiazole INDY (Fig 4).

**Fig 5:**
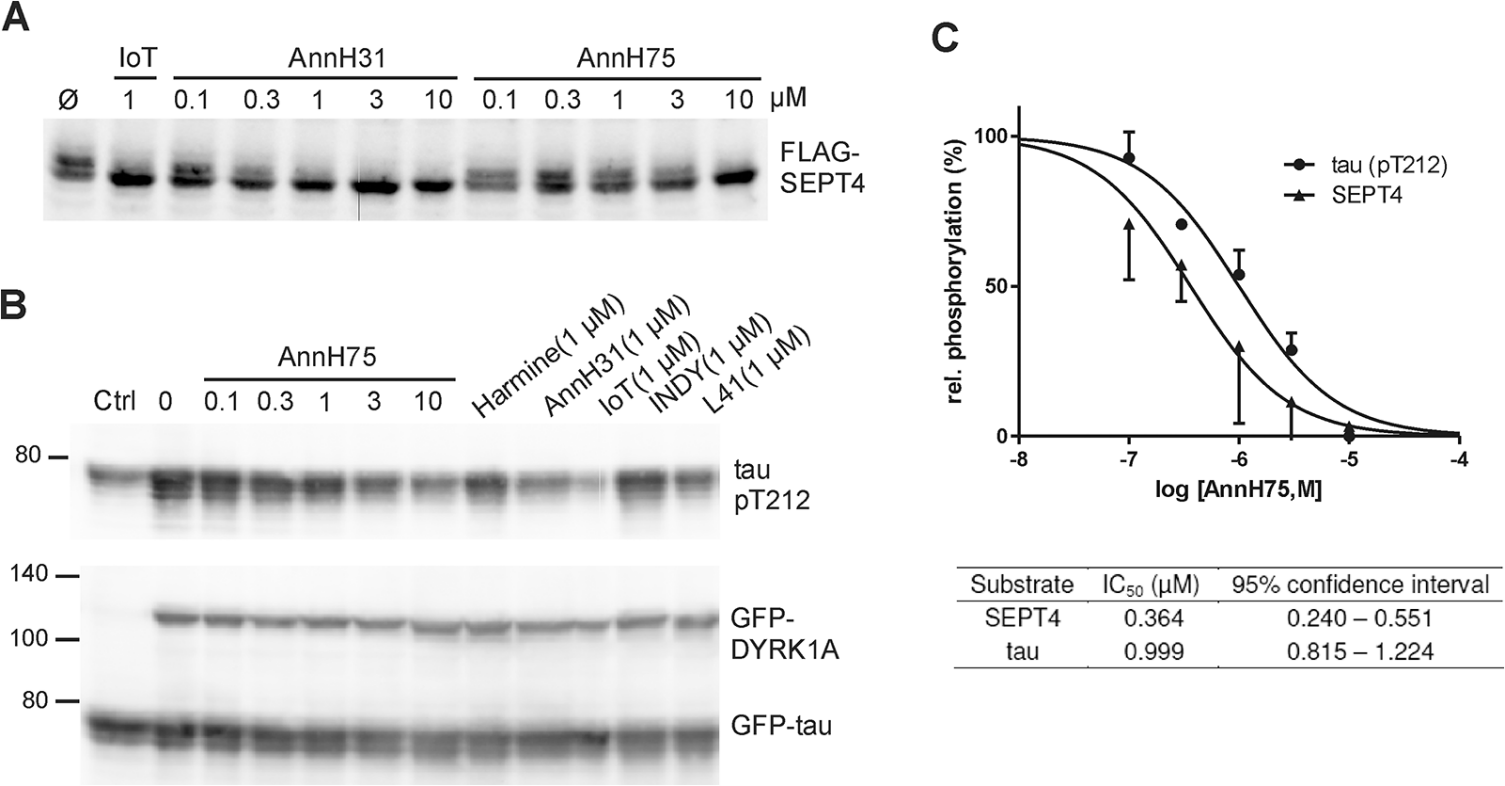
Inhibition of SEPT4 and tau phosphorylation by DYRK1A. **A**, HeLa cells transiently expressing FLAG-SEPT4 were treated with AnnH31 or AnnH75 for 5 h before cells were lysed and analysed by immunoblotting with a FLAG-tag antibody. 5-iodotubercidin (IoT) served as positive control. Relative SEPT4 phosphorylation was calculated as the ratio of the intensities of the phosphorylated upper band and the lower band. **B**, HEK293 cells with constitutive expression of GFP-tau and regulatable expression of GFP-DYRK1A were treated with doxycyclin and the indicated inhibitors for 18 h. Phosphorylation of tau on Thr212 was detected with a phosphospecific antibody. Expression levels of GFP-tau and GFP-DYRK1A were assessed with a GFP antibody. For quantitative evaluation of DYRK1A inhibition, the basal pT212 signal in control cells not treated with doxycyclin (Ctrl) was subtracted from all values. **C**, Quantitative evaluation of three experiments each for SEPT4 and tau. All data were standardized to the level of phosphorylation in cells untreated with inhibitors. Error bars indicate SEM.

We next analysed the phosphorylation of SEPT4 by DYRK1A, which can be detected by means of its reduced electrophoretic mobility [47]. Treatment of HeLa cells expressing FLAG-SEPT4 with AnnH31 and AnnH75 resulted in a concentration-dependent decrease of intensity of the slower migrating band and a concomitantly increased signal of the lower band (Fig 5A). Consistent with the IC_50_ values determined in the *in vitro*-assays (Table 1), AnnH31 reduced SEPT4 phosphorylation more potently than AnnH75.

As a third cell-based assay of DYRK1A inhibition, we measured the phosphorylation of the microtubule-associated protein tau by DYRK1A. The phosphorylation of tau (gene symbol *MAPT*) by DYRK1A has been considered potentially relevant in the early Alzheimer’s disease-like neurodegeneration in Down syndrome and in spontaneous Alzheimer’s disease [10]. In this assay, AnnH75 inhibited DYRK1A activity with an IC_50_ of 1 μM (Fig 5B and 5C). The higher value as compared to the inhibition of SEPT4 phosphorylation (0.36 μM) may be due to the overexpression of DYRK1A in this assay. AnnH31, L41 and 5-iodotubercidin were the best inhibitors in this assay, whereas INDY reduced tau phosphorylation less efficiently. Low cellular potency of INDY has previously been observed in assays of tau phosphorylation and reporter gene activity [30]. Similarly, two other DYRK1A inhibitors with excellent potency and selectivity in biochemical assays require ~1000 higher concentrations to inhibit tau phosphorylation in cellular assays [35–36]. Taken together, our results provide evidence that AnnH31 and AnnH75 can be used as chemical probes to confirm the participation of DYRK1A in cellular phosphorylation events.

### AnnH75 inhibits tyrosine autophosphorylation of DYRK1A

**Fig 6:**
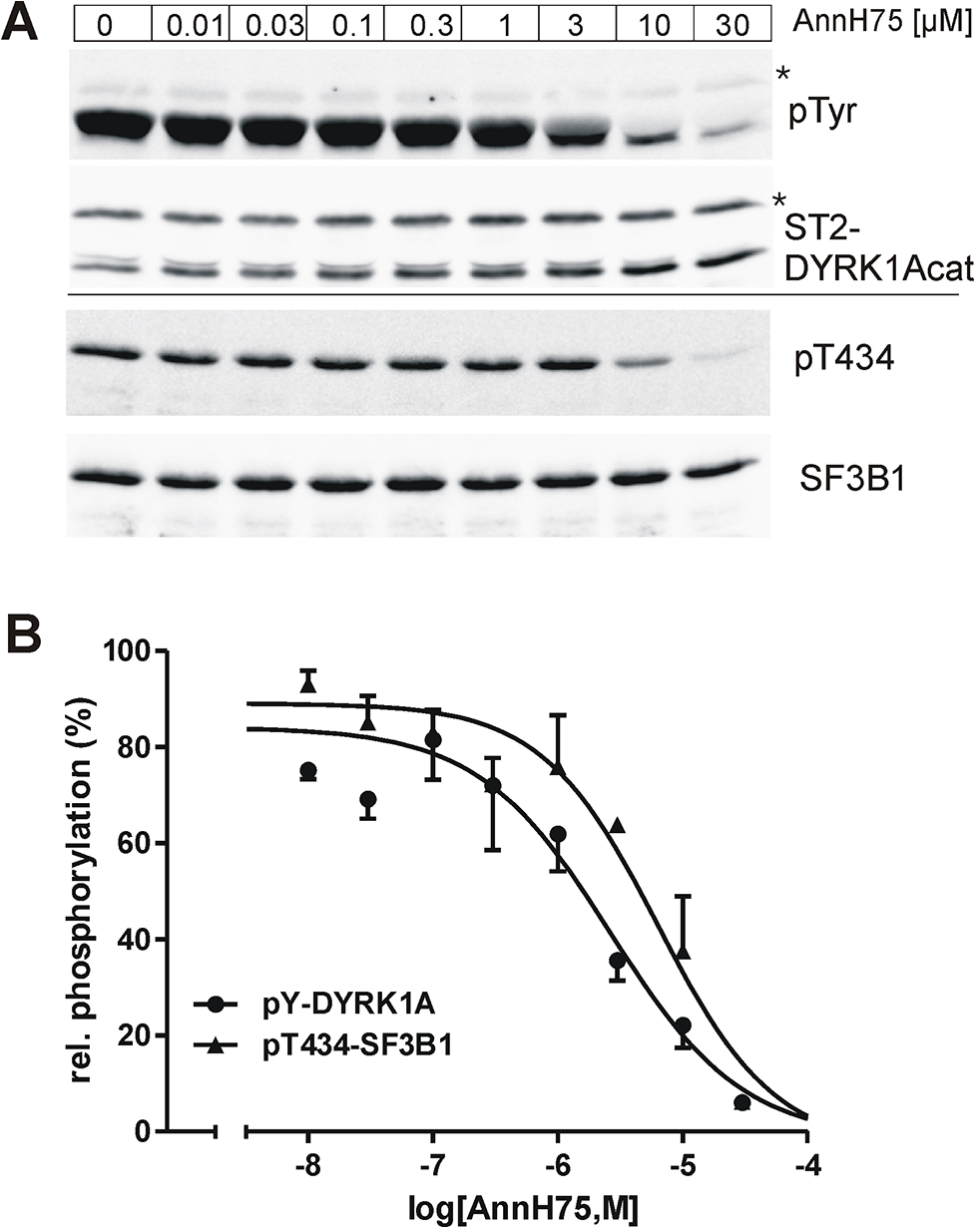
AnnH75 inhibits both threonine and tyrosine kinase activity of DYRK1A. A DYRK1A construct with an N-terminal StrepTag 2 (ST2-DYRK1Acat) was expressed in a cell-free *E*. *coli*-derived expression system. Coupled *in vitro* transcription and translation reactions were incubated for 1 h in the presence of recombinant SF3B1-NT-His_6_ and AnnH75. Phosphorylation of tyrosines in DYRK1A and of Thr434 in SF3B1 was determined by immunoblotting with a phosphotyrosine-specific antibody (pTyr) and a pThr434-specific antibody. **A**, Representative western blots. The asterisks mark unidentified bands. **B**, Quantitative evaluation. Results were normalized to the total amount of DYRK1A or SF3B1, respectively, and are plotted relative to the phosphorylation in the untreated control samples (means +/- SEM, n = 3).

Tyrosine kinase activity of DYRKs has been postulated to exhibit different inhibitor sensitivity than serine/threonine phosphorylation [7]. Therefore we investigated if AnnH75 is a selective inhibitor of the serine/threonine kinase activity of mature DYRK1A or whether AnnH75 also interferes with tyrosine autophosphorylation during translation. To address this question, we used a coupled *in vitro*-transcription-translation system and monitored at the same time the effect of the inhibitor on the tyrosine autophosphorylation of DYRK1A and the phosphorylation of Thr434 in the substrate protein SF3B1 (Fig 6). Under these conditions, AnnH75 inhibited tyrosine autophosphorylation of DYRK1A at concentrations >1 μM. Slightly higher concentrations of AnnH75 were required for the inhibition of threonine phosphorylation in SF3B1. The higher IC_50_ value for SF3B1 phosphorylation as compared to the peptide assay is due to the fact that kinase activity in the *in vitro*-translation mix cannot be assayed under conditions of substrate saturation (as discussed previously [9]). In conclusion, AnnH75 inhibits both the immature and mature form of DYRK1A.

## Conclusions

Chemical probes serve as invaluable small molecule tools to functionally annotate signalling proteins, to understand their role in physiological and pathological processes and to validate them as potential drug targets. Here we report the evaluation of new harmine analogs with regard to their suitability as chemical probes for the protein kinase DYRK1A. To support the conclusion that AnnH31 and AnnH75 meet the key requirements of a useful tool for this purpose, we discuss our results with respect of the “fitness factors” of chemical probes that were proposed by Workman & Collins [6]: selectivity, potency, chemistry and context.

### Selectivity

A key criterion for the use of a kinase inhibitor as a chemical probe is that its selectivity profile is well defined [4,6]. The kinase selectivity profile of AnnH75 compares favourably with that of other DYRK1A inhibitors that have been profiled against an informative panel of kinases (Table 2). However, the cross-reactivity for CLK1/4 and haspin clearly requires consideration when this DYRK1A inhibitors is used in experiments addressing splicing regulation or cell cycle control.

Regarding off-targets outside the protein kinase family, we reached the goal of eliminating the MAO-A inhibitory activity of harmine. AnnH31 displays already 40 fold selectivity for DYRK1A over MAO-A and complies with the requirements for a high quality chemical probe [6]. AnnH75 has no residual effect on MAO-A and is better suited in all applications potentially susceptible to monoamine effects.

The availability of inactive analogs and active probes from a different chemical class can help to prove target-dependency in a biological experiment [4,6]. When AnnH75 is used as a probe to confirm a hypothetical function of DYRK1A or DYRK1B, AnnH79 can serve as a structurally related inactive control compound (Fig 4) and L41 as a potent inhibitor with a different chemotype.

### Biochemical and cellular potency

Several mechanisms can account for discrepancies between inhibitor potency in biochemical and in the cellular context [3,48]. Many DYRK1A inhibitors with adequate potencies in biochemical assays (< 100 nM, as proposed by Workman & Collins [6]) have been described [14], but only few have been shown to inhibit the phosphorylation of a cellular target in a concentration-dependent manner with a practicable potency (< 1–10 μM according to Workman & Collins [6]). In particular, the only known inhibitors with significant selectivity for DYRK1A over CLK1 require micromolar concentrations for half-maximal effects in cellular assays, in spite of low nanonmolar potencies in biochemical assays [35–36]. We verified the cellular efficacy of AnnH75 with three different substrates of DYRK1A and provide direct comparisons with established DYRK1A inhibitors. Taken together, the concentration-dependent effects of the chemical probes (AnnH31 or AnnH75) support the hypothesis that a molecular mechanism depends on a certain target (in this case DYRK1A).

### Chemistry (structure, solubility, stability, permeability)

The procedures for the synthesis and the structural characterization of the new β-carboline compounds will be fully described in a separate manuscript, and the structure of the AnnH75/DYRK1A cocrystal has been solved (PDB ID 4YU2). The physicochemical properties of AnnH31 and AnnH75 comply with Lipinski’s “rules of 5”, which predict oral bioavailability of drugs [49]) (Table B in S1 File). The efficacy in cell-based assays indicates that AnnH31 and AnnH75 are membrane-permeable and sufficiently soluble in aqueous media. Moreover, we found that the new substances were still active after prolonged incubation at 37°C in cell culture medium (Fig. A in S1 File). It remains to be examined whether AnnH31 and AnnH75 are metabolically stable in animal experiments.

### Context

It is to be emphasized that the appropriateness of a chemical probe depends on the hypothesis under investigation and our knowledge of the biological context [6]. Thus, the characterization of the effects of AnnH31 and AnnH75 on on-target and off-target pathways will help to establish the suitability of the probes in a specific context. The interpretation of the phenotypic effects of a DYRK1 inhibitor is strengthened if the inhibition of kinase activity or the effect on a downstream pathway can be monitored. For example, the indole derivative ID-8 was found to maintain human stem cells in an undifferentiated state in chemically defined xeno-free medium and was proposed to act as a DYRK inhibitor [50]. This is a very important observation, although ID-8 has not yet been scrutinized according to the fitness criteria of a chemical probe. The presumed role of DYRK1A in this context could be further corroborated by using AnnH75 and L41 as potent, specific and well characterized DYRK1 inhibitors in parallel with inactive chemical analogs, and by assessing cellular DYRK1A inhibition by ID-8.

## Materials and Methods

### Antibodies

Mouse monoclonal antibodies against phosphotyrosine (PY99; Santa Cruz Biotechnology, Santa Cruz, CA, USA), SF3B1 (SAP155; MBL, Nagoya, Aichi, Japan), and FLAG-tag (FLAG BioM2, Sigma Aldrich GmbH, München, Germany), goat antibody against GFP (Rockland Immunochemicals Inc., Gilbertsville, PA, USA), a rabbit antibody for phosphorylated Thr212 in the tau protein (#44740G, Invitrogen, Camarillo, USA) as well as *Strep*-Tactin HRP conjugate (IBA, Göttingen, Germany) were purchased commercially. The rabbit antibody for detecting phosphorylated Thr434 in SF3B1 has been described [51].

### Chemicals

Harmine was obtained from Fluka, Buchs, Switzerland and 5-iodotubercidin from Tocris Bioscience, Minneapolis, MN, USA. A report on the synthesis of the harmine analogues will be presented elsewhere. All inhibitors were dissolved in dimethylsulfoxide (DMSO), except for harmine which was dissolved in ethanol. Further dilutions of all inhibitors were prepared in water to get final concentrations of 3% DMSO in *in vitro* translation reactions and cell assays or 1% DMSO in kinase activity assays if not indicated otherwise.

### Preparation of recombinant kinases

Bacterial expression plasmids for GST-DYRK1A-ΔC and GST-DYRK2 have been described earlier [52–53]. To generate the GST-DYRK1Bcat, GST-HIPK2cat and GST-CLK1cat expression vectors, human cDNAs encoding the catalytic domain of DYRK1B (amino acids 87–451 of the reference sequence NP_004705), HIPK2 (amino acids 180–533 of the reference sequence NP_00110671) or CLK1 (amino acids 141–484 of the reference sequence NP_004062) were inserted into pGEX-2TK vector *via* engineered *Bam*HI and *Eco*RI sites. Expression in logarithmically growing *E*. *coli* culture was performed for 3 h at 37°C (GST-DYRK1A-ΔC and GST-DYRK2), 3 h at 26°C (GST-HIPK2cat), over night at 28°C (GST-CLK1cat) or over night at room temperature (GST-DYRK1Bcat). GST fusion proteins were partially purified by affinity adsorption to glutathione-Sepharose and stored in the elution buffer (50 mM Tris-HCl pH 8.0, 10 mM reduced glutathione) at -80°C until use.

### MAO-A assay

Inhibition of monoamine oxidase A (MAO-A) was determined with the MAO-GLO Assay kit from Promega according to the manufacturer’s instructions using 12 μU human recombinant MAO-A and 25 μM MAO-A substrate in a total volume of 20 μL.

### Kinase assays

Compounds were screened for inhibitory effects against GST-DYRK1A-ΔC, GST-DYRK1Bcat, GST-DYRK2, GST-CLK1cat or GST-HIPK2cat using the Kinase-GLO Luminescent Kinase Assay from Promega. Assays were performed in a total volume of 10 μL in kinase-buffer (25 mM Hepes pH 7.4, 0.5 mM DTT, 5 mM MgCl_2_, 5 μM ATP) with appropriate peptide substrates (20 μM DYRKtide for the DYRKs and 100 μM DYRKtide for HIPK2, RRRFRPASPLRGPPK; or 100 μM RS domain-derived peptide for CLK1, GRSRSRSRSR). Assays were started with this amount of a kinase preparation that consumed approximately 90% of the ATP in the assay (linear range of the kinase titration curve). The reactions were run at room temperature for 30 min before 10 μL Kinase-GLO reagent was added. After incubation at room temperature for additional 10 min, luminescence was recorded for 1 s.

Radioactive kinase assays were used to determine IC_50_ values of selected DYRK1A inhibitors. GST-DYRK1A-ΔC was incubated with 100 μM DYRKtide, 100 μM ATP, 5 μCi [γ-^33^P]-ATP and variable concentrations of the inhibitors for 5 min at 30°C. Each sample was assayed in triplicate. Reactions were stopped by pipetting aliquots of the reaction mix onto P81 phosphocellulose paper and immediate immersion in 5% phosphoric acid. Phosphocellulose papers were washed at least 5 times and radioactivity was measured by scintillation counting. Background values from samples incubated without kinase were subtracted and phosphate incorporation was normalized to the control samples incubated without inhibitor. IC_50_ values were calculated with the help of the GRAPHPAD PRISM 5.0 program (GraphPad Software, La Jolla, CA, USA) after automatic outlier elimination.

The profiling of AnnH75 against a 300-kinase panel was performed by ProQinase (Freiburg, Germany) using radiometric protein kinase assays in a duplicate measurement. Gini coefficients were calculated and the Lorenz curve (Fig 3B) was plotted by applying the Microsoft Excel spreadsheet provided by Graczyk [44].

### Cell viability assay

Cytotoxicity was evaluated in HeLa, PC12 [54], originally purchased from Clontech Laboratories Inc., Cat. No. 630912) and SH-SY5Y cells [17] after 3 days of incubation with the test compounds by using a tetrazolium dye assay (XTT assay, AppliChem GmbH, Darmstadt, Germany).

### *In vitro* translation assay

The PURExpress In Vitro Protein Synthesis Kit (New England Biolabs, Beverley, MA, USA), which is a reconstituted *E*. *coli*-based *in vitro* transcription-translation system, was used to express a DYRK1A construct comprising the catalytic domain of DYRK1A fused to an N-terminal Strep-tag II sequence. Reactions were run in a total volume of 10 μL with 10 ng/μL pET-ST2-DYRK1A [9] and 80 ng/μL SF3B1-NT-His6 as a substrate [51] at 37°C for 1 h. AnnH75 or solvent control (3% DMSO) was added as indicated. Reactions were stopped by adding 2x Laemmli sample buffer and 5 mM EDTA. Tyrosine autophosphorylation of DYRK1A and phosphorylation of SF3B1 on Thr434 was analysed by western blotting. Band intensities were quantified using the AIDA Image Analyzer 5.0 program (Raytest, Straubenhardt, Germany).

### Cellullar assays

HeLa cells were seeded in 6-well plates and transfected (400 ng/well) with expression vectors for GFP-SF3B1NT (containing amino acids 1–492 of human SF3B1 [51]) or FLAG-Septin4 [47] (500 ng/well) using FuGENE HD (Promega, Mannheim, Germany). After 24 h, SF3B1 expressing cells were treated with inhibitors for 18 h. SEPT4 expressing cells were cultivated for 48 h before they were incubated with the inhibitors for 5 h. Tau phosphorylation was assayed using a HEK293 subclone with regulatable expression of GFP-DYRK1A and constitutive expression of GFP-tau (HEK293-tau-Dyrk1A) that was kindly provided by Dr. Matthias Engel (Department of Pharmaceutical and Medicinal Chemistry, Saarland University, Saarbrücken, Germany) [55]. Expression of GFP-DYRK1A was induced with 2 μg/mL doxycyclin before cells were treated with the inhibitors for 20 h.

In all assays, cells were lysed with 100 μL SDS lysis buffer (20 mM Tris HCl pH 7.4, 1% SDS), samples were sonicated and cleared by centrifugation before SDS-PAGE and immunoblotting. Immunoreactivities were detected by enhanced chemiluminescence using HRP-coupled secondary antibodies and quantified using the AIDA Image Analyzer 5.0 program (Raytest, Straubenhardt, Germany). Relative phosphorylation of SF3B1 and tau was calculated by normalisation to total protein levels. To calculate relative DYRK1A activity in tau assays, the basal pT212 signal in control cells not treated with doxycyclin was subtracted from all values, and the phosphorylation in DYRK1A expressing cells not treated with inhibitors was set to 100%. SEPT4 phosphorylation was determined as the ratio of intensities of the phosphorylated upper band to the lower band. Curve fitting for IC_50_ determination was done with the help of the GRAPHPAD PRISM 5.0 program (GraphPad Software, La Jolla, CA, USA).

## Supporting Information

S1 File Table A. Kinome profiling of AnnH75. **Table B.** Drug like properties of AnnH31 and AnnH75. **Fig A.** Inhibition of DYRK1A and MAO-A by selected β-carbolines. **Fig B.** Predicted binding mode of AnnH75 at DYRK1B (homology model). **Fig C.** Inhibition of DYRK1A by β-carbolines after prolonged incubation in aqueous solution. **Fig D.** Chemical structures of DYRK inhibitors. **Supplementary Methods.** Computational methods applied for docking studies.
(PDF)
