# Supplementary material for: Selectivity Profiling and Biological Activity of Novel β-Carbolines as Potent and Selective DYRK1 Kinase Inhibitors
Source: PLoS One. 2015 Jul 20;10(7):e0132453. doi: 10.1371/journal.pone.0132453 (PMC4508061; doi:10.1371/journal.pone.0132453)
Supplement: S1 File — Table B. Drug like properties of AnnH31 and AnnH75. Fig A. Inhibition of DYRK1A and MAO-A by selected β-carbolines. Fig B. Predicted binding mode of AnnH75 at DYRK1B (homology model). Fig C. Inhibition of DYRK1A by β-carbolines after prolonged incubation in aqueous solution. Fig D. Chemical structures of DYRK inhibitors. Supplementary Methods. Computational methods applied for docking studies. (PDF) [file pone.0132453.s001.pdf]

Supporting Information

Selectivity profiling and biological activity of novel β-carbolines as potent and selective DYRK1 kinase inhibitors

Katharina Rüben[1], Anne Wurzlbauer[2], Agnes Walte[1], Wolfgang Sippl[3], Franz Bracher[2], Walter Becker[1*]

[1] Institute of Pharmacology and Toxicology, RWTH Aachen University, Aachen, Germany
[2] Department of Pharmacy - Center for Drug Research, Ludwig-Maximilians-University, Munich, Germany
[3] Institute of Pharmacy, Martin-Luther-University Halle-Wittenberg, Halle, Germany

*Correspondence should be addressed to: wbecker@ukaachen.de

TABLE OF CONTENTS

Table A: Kinome profiling of AnnH75

Table B: Drug like properties of AnnH31 and AnnH75

Figure A: Inhibition of DYRK1A and MAO-A by selected β-carbolines

Figure B: Predicted binding mode of AnnH75 at DYRK1B

Figure C: Inhibition of DYRK1A by β-carbolines after prolonged incubation in aqueous solution

Figure D: Chemical structures of DYRK inhibitors

Supplementary Methods: Computational methods applied for docking studies

Table A. Kinome profiling of AnnH75

Kinase	residual activity at 1 µM (% of control) Exp. 1	Exp. 2	Mean
ABL1	96	93	95
ABL2	97	94	96
ACK1	105	90	97
ACV-R1	93	94	93
ACV-R1B	90	103	97
ACV-R2A	92	101	97
ACV-R2B	103	92	97
ACV-RL1	91	91	91
AKT1	104	101	102
AKT2	120	92	106
AKT3	107	100	104
ALK	100	96	98
AMPK-alph1	98	86	92
ARK5	103	86	94
ASK1	106	101	104
Aurora-A	94	84	89
Aurora-B	103	105	104
Aurora-C	97	97	97
AXL	96	93	95
BLK	103	98	100
BMPR1A	89	88	89
BMX	87	72	79
B-RAF	103	106	105
BRK	107	88	97
BRSK1	103	111	107
BTK	98	94	96
CAMK1D	98	106	102
CAMK2A	85	72	79
CAMK2B	93	91	92
CAMK2D	92	82	87
CAMK4	100	122	111
CAMKK1	84	95	89
CAMKK2	88	83	86
CDC42BPA	74	79	76
CDC42BPB	99	89	94
CDK1/CycA2	80	77	79
CDK1/CycB1	81	80	81
CDK1/CycE1	96	91	93
CDK2/CycA2	86	91	88
CDK2/CycE1	97	96	97
CDK3/CycE1	94	95	94
CDK4/CycD1	111	89	100
CDK4/CycD3	90	88	89
CDK5/p25NCK	99	94	96
CDK5/p35NCK	96	90	93
CDK6/CycD1	95	96	95
CDK7/CycH/MAT1	95	104	100
CDK8/CycC	20	17	18
CDK9/CycK	51	53	52
CDK9/CycT1	83	86	85
CHK1	100	101	100
CHK2	103	102	102
CK1-alpha1	88	91	90
CK1-delta	72	71	71
CK1-epsilon	76	79	78
CK1-gamma1	92	92	92
CK1-gamma2	82	82	82
CK1-gamma3	94	91	93
CK2-alpha1	96	95	96
CK2-alpha2	101	100	101
CLK1	1	-2	0
CLK2	45	42	43
CLK3	75	73	74
CLK4	-1	0	-1
COT	90	86	88
CSF1-R	111	101	106
CSK	101	87	94
DAPK1	93	90	91
DAPK2	102	91	97
DAPK3	98	97	98
DCAMKL2	108	86	97
DDR2	97	97	97
DMPK	102	101	101
DNA-PK	63	63	63
DYRK1A	8	8	8
DYRK1B	8	7	8
DYRK2	69	68	68
DYRK3	60	55	57
DYRK4	97	92	94
EEF2K	106	91	98
EGF-R	109	98	104
EIF2AK2	99	91	95
EIF2AK3	97	101	99
EPHA1	89	95	92
EPHA2	91	95	93
EPHA3	107	103	105
EPHA4	101	96	99
EPHA5	82	87	84
EPHA7	115	100	107
EPHA8	85	92	89
EPHB1	90	89	89
EPHB2	100	99	99
EPHB3	101	90	96
EPHB4	97	94	95
ERBB2	112	92	102
ERBB4	89	91	90
ERK1	94	80	87
ERK2	93	81	87
ERK7	62	57	59
FAK	105	101	103
FER	97	90	93
FES	99	87	93
FGF-R1	98	96	97
FGF-R2	88	83	85
FGF-R3	112	103	107
FGF-R4	100	95	98
FGR	98	103	101
FLT3	71	70	70
FRK	94	95	94
FYN	96	88	92
GRK2	96	88	92
GRK3	94	90	92
GRK4	54	45	49
GRK5	98	92	95
GRK6	104	93	98
GRK7	108	99	103
GSG2 (Haspin)	5	6	5
GSK3-alpha	89	74	81
GSK3-beta	100	92	96
HCK	106	97	101
HIPK1	95	88	91
HIPK2	83	91	87
HIPK3	99	76	88
HIPK4	88	89	88
HRI	98	104	101
IGF1-R	81	86	83
IKK-alpha	61	58	60
IKK-beta	94	89	91
IKK-epsilon	93	97	95
INS-R	92	91	92
INSR-R	97	97	97
IRAK1	97	92	95
IRAK4	96	89	92
ITK	103	87	95
JAK1	94	93	93
JAK2	102	96	99
JAK3	100	101	101
JNK1	108	90	99
JNK2	96	104	100
JNK3	78	86	82
KIT	86	79	82
LCK	93	90	91
LIMK1	55	59	57
LIMK2	86	102	94
LRRK2	88	77	83
LTK	110	89	99
LYN	83	72	77
MAP3K1	99	95	97
MAP3K10	91	91	91
MAP3K11	100	96	98
MAP3K7	92	90	91
MAP3K9	95	105	100
MAP4K2	92	94	93
MAP4K4	89	81	85
MAP4K5	84	84	84

MAPKAPK2	89	80	85
MAPKAPK3	102	87	94
MAPKAPK5	92	85	89
MARK1	103	99	101
MARK2	96	100	98
MARK3	103	97	100
MARK4	105	103	104
MATK	103	102	102
MEK1	65	79	72
MEK2	40	34	37
MEKK2	105	104	105
MEKK3	101	92	97
MELK	91	83	87
MERTK	97	86	91
MET	101	99	100
MINK1	94	90	92
MKK6[a]	89	111	100
MKNK1	101	105	103
MKNK2	105	79	92
MST1	92	90	91
MST2	106	95	101
MST3	111	98	104
MST4	97	88	92
mTOR	86	94	90
MUSK	94	108	101
MYLK	93	91	92
MYLK2	88	92	90
MYLK3	94	94	94
NEK1	107	95	101
NEK11	94	95	94
NEK2	85	96	90
NEK3	87	96	91
NEK4	103	93	98
NEK6	102	106	104
NEK7	96	91	93
NEK9	95	95	95
NIK	88	87	87
NLK	86	98	92
p38-alpha	88	85	86
p38-beta	107	104	105
p38-delta	91	87	89
p38-gamma	86	86	86
PAK1	104	102	103
PAK2	94	117	105
PAK3	89	101	95
PAK4	94	93	94
PAK6	95	91	93
PAK7	95	84	90
PASK	74	73	74

PBK	97	92	94
CDK16/CycY	92	89	90
PDGFR-alpha	107	97	102
PDGFR-beta	93	84	88
PDK1	100	98	99
PHKG1	84	99	91
PHKG2	92	96	94
PIM1	43	39	41
PIM2	120	95	107
PIM3	44	43	44
PKA	101	109	105
PKC-alpha	105	117	111
PKC-beta1	76	109	93
PKC-beta2	88	128	108
PKC-delta	101	109	105
PKC-epsilon	30	48	39
PKC-eta	78	79	79
PKC-gamma	105	110	108
PKC-iota	90	115	102
PKC-mu	100	101	100
PKC-nu	92	82	87
PKC-theta	108	118	113
PKC-zeta	70	124	97
PLK1	98	87	93
PLK3	97	94	96
PRK1	91	97	94
PRK2	87	89	88
PRKD2	87	93	90
PRKG1	93	81	87
PRKG2	83	86	85
PRKX	90	71	80
PYK2	114	106	110
RAF1[a]	98	99	99
RET	89	88	89
RIPK2	92	96	94
RIPK5	96	86	91
ROCK1	77	69	73
ROCK2	81	77	79
RON	97	95	96
ROS	94	93	94
RPS6KA1	68	59	63
RPS6KA2	101	92	97
RPS6KA3	105	98	101
RPS6KA4	99	90	95
RPS6KA5	100	92	96
RPS6KA6	98	97	98
S6K	85	85	85
S6K-beta	98	106	102
SAK	104	119	112

SGK1	94	88	91
SGK2	99	99	99
SGK3	109	107	108
SLK	103	94	99
SNARK	96	94	95
SNF1LK2	93	89	91
SNK	95	89	92
SRC	95	95	95
SRMS	95	89	92
SRPK1	106	115	111
SRPK2	110	102	106
STK17A	92	86	89
STK23	94	92	93
STK25	94	98	96
STK33	87	86	87
STK39	87	83	85
SYK	106	97	101
TAOK2	104	93	98
TAOK3	104	101	103
TBK1	95	93	94
TEC	102	84	93
TGFB-R1	104	105	104
TGFB-R2	87	80	83
TIE2	91	78	84
TLK1	96	85	91
TLK2	111	106	108
TRK-A	83	86	84
TRK-B	85	59	72
TRK-C	92	83	87
TSF1	99	95	97
TSK2	92	93	92
TSSK1	88	82	85
TTK	95	87	91
TXK	92	97	94
TYK2	99	92	96
TYRO3	97	89	93
VEGF-R1	92	85	89
VEGF-R2	96	100	98
VEGF-R3	99	87	93
VRK1	88	89	89
WEE1	92	109	100
WNK1	102	95	98
WNK2	94	101	98
WNK3	95	92	94
YES	86	80	83
ZAK	98	89	94
ZAP70	98	95	97

The profiling of AnnH75 against 300 protein kinases was performed by ProQinase (Freiburg, Germany) using radiometric protein kinase assays. Kinases inhibited by >90% or 50-90% are highlighted by shading in red or yellow, respectively.

[a] Constitutively active point mutants were used for assays of MKK6 (S207D/T211D) and Raf (Y340D/Y341D).

Table B. Drug like properties of AnnH31 and AnnH75

	Rules of 5[a]	AnnH31	AnnH75
hydrogen bond donors	≤5	0	0
hydrogen bond acceptors	≤ 10	4	4
molecular mass	≤ 500	251	271
logP (octanol-water partition coefficient) [c]	≤ 5	2.5	2.5
	**Further criteria**[b]		
total polar surface area [c]	≤ 140 Å^2	50.9	50.9
rotatable bonds	≤ 10	2	2

[a] Lipinski, C.A., Lombardo, F., Dominy, B.W., Feeney, P.J. (1997) Experimental and computational approaches to estimate solubility and permeability in drug discovery and development settings. *Adv. Drug Delivery Rev. 23*, 4-25.
[b] Veber, D.F., Johnson, S.R., Cheng, H.Y., Smith, B.R., Ward, K.W., Kopple, K.D. (2002) Molecular properties that influence the oral bioavailability of drug candidates. *J. Med. Chem. 6*, 45, 2615-23.
[c] estimated using the Molinspiration logP engine. Only for 3.5% of structures logP is predicted with error > 1.0 (www.molinspiration.com).

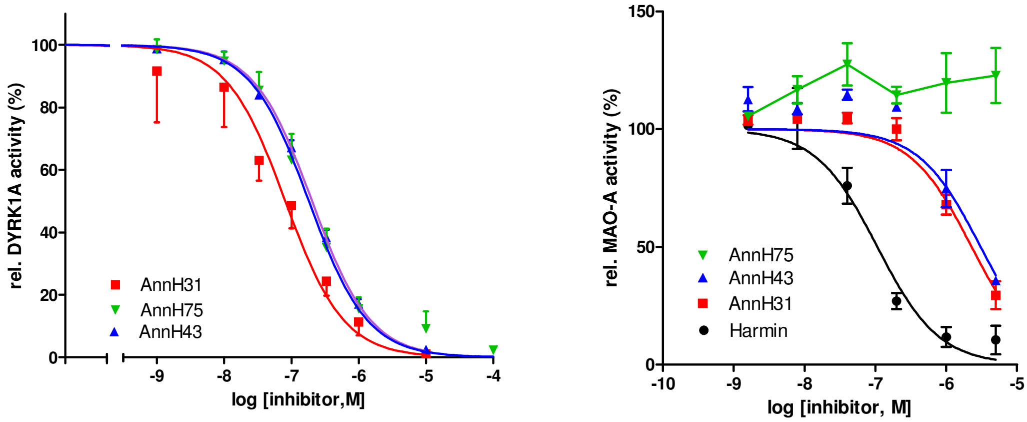

Fig. A. Inhibition of DYRK1A and MAO-A by selected β-carbolines.
The graphs show the concentration response curves from which the IC_{50} values given in Table 2 were calculated. Kinase activity of DYRK1A was determined by radiometric assays using the peptide DYRKtide as a substrate. MAO-A activity was measured with the help of the MAO-GLO assay kit (Promega). IC_{50} values were calculated by curve fitting with the help of the GRAPHPAD PRISM 5.0 program after automatic outlier elimination. The graphs present the results of three to five independent assays of each inhibitor.

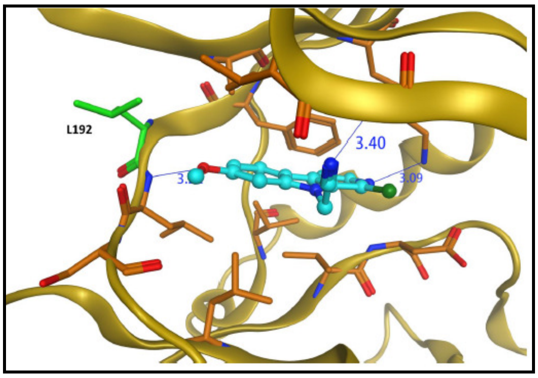

Fig. B. Predicted binding mode of AnnH75 at DYRK1B (homology model).
The inhibitor is colored cyan and DYRK1B is shown as yellow ribbon. Only interacting amino acid residues in the ATP binding pocket are shown for clarity. The residue differing from DYRK1A (L192) is colored green. The distances of the two hydrogen bonds and the distance between the cyano group and the glycine of the P-loop are given in Angstrom.

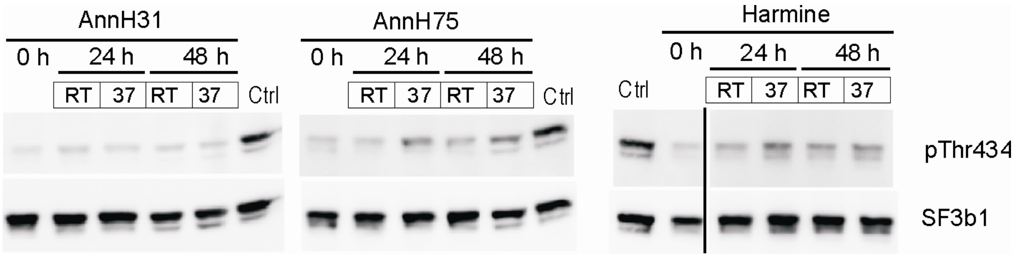

Fig. C. Inhibition of DYRK1A by β-carbolines after prolonged incubation in aqueous solution.
The indicated compounds were dissolved in cell culture medium at a concentration of 10 µM and incubated at room temperature (~20°C) or in the incubator at 37°C for 24 h or 48 h. HeLa cells expressing GFP-SF3B1 were treated with the pre-incubated inhibitors or with freshly diluted compounds (0 h) as indicated for 24 h at a final concentration of 1 µM. Control cells remained untreated (Ctrl). Phosphorylation of SF3B1 was detected by western blot analysis with an antibody specific for pThr434.

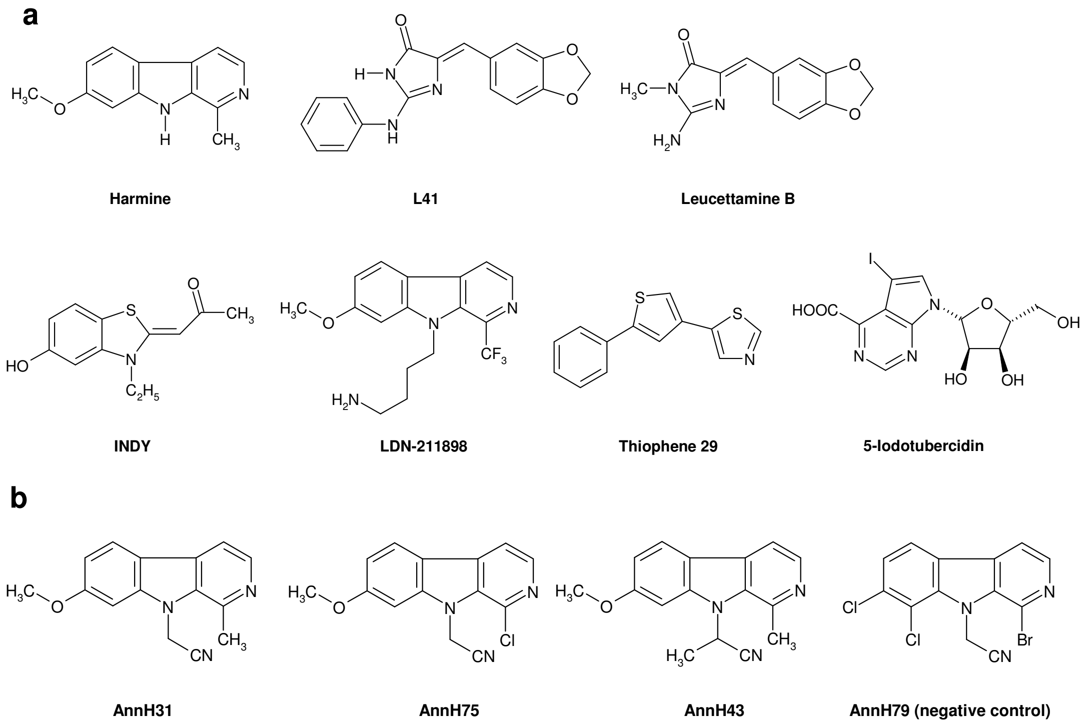

Fig. D. Chemical structures of DYRK inhibitors.
a, Previously known DYRK inhibitors
b, Harmine derivatives from the present investigation

Supplementary Methods: Computational methods applied for docking studies.

All calculations were performed on a Pentium IV 2.2 GHz based Linux cluster (20 CPUs). Program GOLD5.1 (Jones et al. 1997) was used for ligand docking, whereas the calculation of all molecular descriptors and the analysis of the docking results were carried out in MOE20012.10 (Chemical Computing Group). The ligands were constructed using MOE2013.10 and energy minimized using the MMFF94 force field using a convergence criterion of 0.1 kcal/mol. The inhibitor was docked into the binding pocket of the X-ray structure of DYRK1A (PDB code 3ANR complexed with harmine which represents the active form of DYRK1A), DYRK2 (PDB code 4AZF complexed with leucettine L41, active form of DYRK2), CLK1 (PDB code 2VAG complexed with KH-CB19, active form of CLK1). The cocrystallized inhibitor was defined as centre of the enclosing box used for docking with a radius of 15 Å.

To test whether the used docking protocol is suitable for DYRK1A docking the cocrystallized inhibitor was redocked into DYRK1A. Using GoldScore as scoring function an RMSD value of 0.39 Å was derived for the top-ranked docking pose of harmine. For all docked inhibitors the GoldScore was calculated and analyzed. In a similar way docking to the DYRK2 crystal structure was carried out.

Docking to CLK1 was carried out using the crystal structure in complex with the small molecule inhibitor KH-CB19 which has a similar size and shape as harmine. Two hydrogen bonds are observed between inhibitor and kinase similar to the DYRK1A complex: one to the hinge region residue L244 and one to K191. To test whether the used docking protocol is suitable for CLK1 docking the co-crystallized inhibitor was redocked into the ATP binding pocket. Using GoldScore as scoring function an RMSD value of 0.61 Å was derived for the top-ranked docking pose of KH-CB19.

Since there is no X-ray structure available for DYRK1B and HIPK2, homology models were generated using the program MODELLER. It is a program for comparative protein structure modeling by satisfaction of spatial restraints (Sali et al 1993). The human HIPK2 (Q9H2X6), CLK4 (Q9HAZ1), and DYRK1B (Q9Y463) sequences were taken from Uniprot and a BLAST search was carried out using the protein data bank database for identification of a template structure. DYRK1A was identified as suitable template sharing about 40% overall sequence identity with HIPK2 and about 80% overall sequence identity with DYRK1B. The crystal structure of DYRK1A complexed with harmine (PDB ID 4ANR) was chosen as template for DYRK1B and HIPK2. The same docking protocol as used for DYRK1A was used in case of the homology models.

Jones, G.; Willett, P.; Glen, R.C.; Leach, A.R.; Taylor, R. (1997) Development and validation of a genetic algorithm for flexible docking. *J. Mol. Biol. 267*, 727–748

Sali, A., and Blundell, T.L. (1993) Comparative protein modelling by satisfaction of spatial restraints. *J. Mol. Biol. 234*, 779-815
